# Supplementary material for: Neural circuits in the mouse retina support color vision in the upper visual field
Source: Nat Commun. 2020 Jul 13;11:3481. doi: 10.1038/s41467-020-17113-8 (PMC7359335; doi:10.1038/s41467-020-17113-8)
Supplement: Supplementary file 1 — Supplementary Information [file 41467_2020_17113_MOESM1_ESM.pdf]

SUPPLEMENTARY INFORMATION

Neural circuits in the mouse retina support color vision in the upper visual field

Klaudia P. Szatko, Maria M. Korympidou, Yanli Ran, Philipp Berens, Deniz Dalkara, Timm Schubert, Thomas Euler, Katrin Franke

- Supplementary Discussion
- Supplementary Methods
- Supplementary Figures
- Supplementary References

SUPPLEMENTARY DISCUSSION

In the following, we discuss three observations made while recording light-evoked responses from cones in the retinal whole-mount preparation.

First, our data suggest that the recorded glutamate signals in the OPL depict cone, but not rod signals. First, our functionally defined ROIs formed regular mosaics resembling that of the anatomical cone array [1]. Second, the ROIs co-localized with anatomically identified cone terminals. Third, we did not observe any green-sensitive hyperpolarizing responses upon center stimulation in the ventral retina, as would be expected if glutamatergic output of rod axon terminals contributed to the recorded signals. Why we did not pick up rod signals may be because of differences in the number of vesicles released [2] and number of ribbon synapses (e.g. [3]) between rods and cones. As a result, the amount of glutamate released by individual rod axon terminals may be below the detection threshold of iGluSnFR.

Second, a fraction of cones in our dataset did not exhibit antagonistic surround responses; this was more often the case for dorsal than ventral cones. It is unlikely that cone-to-cone differences in HC input contribute to the strong variations in surround strengths, as HCs form highly stereotypic connections with each cone's axon terminal (e.g. [4,5]). Previous studies have demonstrated that the strength of HC feedback depends on functional cone properties, such as membrane potential [6] and adaptational state (e.g. [7]), which might vary between cells. We controlled for experimental parameters such as temperature and scan field size, while other parameters like biosensor expression and therefore laser power applied could have somewhat varied across recording fields and/or retinal locations. It is conceivable that these factors affected surround strengths in cone RFs.

Third, most UV-sensitive cones in both ventral and dorsal retina exhibited decreases in glutamate release to both center and surround UV stimulation. This was not the case for green-sensitive cones, which showed an increase in glutamate release when presenting a UV surround annulus. The effect observed in UV-cones could be explained by lateral signal spread due to specific cone-cone coupling [8,9] between UV-cones. Such differential coupling between different cone types has been identified in the primate retina [10]; however, evidence for a similar mechanism in the mouse retina is missing. Alternatively, the sign-conserving surround response of UV-cones might be related to the higher sensitivity of S- compared to M-opsin expressing cones [11]. Specifically, light arising from the UV surround stimulus might scatter within the retina, which may be sufficient to drive S-opsin but not M-opsin expressing cones.

SUPPLEMENTARY METHODS

Linear Mixed-Effects Models

We used a Linear Mixed-Effects Model to analyze the difference between center and surround spectral contrast (SC). This allowed to incorporate a random effect term accounting for the fact that not all ROIs with a center response displayed a surround response (partially paired data).

$$y = X\beta + Zu + \epsilon \quad (1)$$

Here, y is the dependent variable SC, β is the coefficient vector for the fixed effects (e.g. center/surround and retinal location), **u** is the coefficient vector of random effects (here: cell ID), ϵ is an unknown vector of random errors and X and Z are known design matrices relating the observations y to β and ϵ, respectively.

We used the `lmerTest`-package (version 3.0.1) for R to implement the model and perform statistical testing [12].

<u>Center and surround SC for OPL recordings</u>

For cones in OPL recordings (Fig. 2b, c), we modeled SC as a function of center and surround ("cent_surr") and cell ID.

```
SC ~ cent_surr + (1|cell_id)                                    (2)
```

For dorsal scan fields, the resulting model was fit using n=922 observations, with n=689 paired observations in group cell_id. Running an ANOVA on the model yielded the following results:

Type III Analysis of Variance Table with Satterthwaite's method

	Sum Sq	Mean Sq	NumDF	DenDF	F value	Pr(>F)
cent_surr	377.62	377.62	1	920	3.7622	0.05273

The p-value ~0.05 (effect size=0.37, s.d. error=0.38) indicates that for dorsal cones center SC is at the threshold of significance.

For ventral scan fields, the resulting model was fit using n=2,181 observations, with n=1,344 paired observations in group cell_id. Running an ANOVA on the model yielded the following results:

Type III Analysis of Variance Table with Satterthwaite's method

	Sum Sq	Mean Sq	NumDF	DenDF	F value	Pr(>F)
cent_surr	1730.5	1730.5	1	2179	178.8	2.2e-16

The model results indicate that for ventral cones center SC is significantly different from surround SC (effect size=-0.63, s.d. error=0.08).

<u>Center and surround SC for IPL and GCL recordings</u>

For ROIs in IPL (Fig. 4b) and GCL (Fig. 6b) recordings, we modeled SC with factors cent_surr and retinal location ("bin_num") as well as their interaction and cell ID as random effect.

```
SC ~ cent_surr * ret_loc + (1|cell_id)                          (3)
```

For IPL ROIs, the resulting model was fit using *n*=6,143 observations, with *n*=3,188 observations in group cell ID. Running an ANOVA on the model yielded the following results:

```
Type III Analysis of Variance Table with Satterthwaite's method
               Sum Sq  Mean Sq   NumDF  DenDF   F value   Pr(>F)
cent_surr      384.14  384.14    1      6139    2863.77   <2e-16
bin_number     23.68   23.68     1      6139    2863.77   <2e-16
cent_surr:bin  264.73  264.73    1      6139    2863.77   <2e-16
```

The model results indicate that for IPL ROIs there is a significant interaction between center and surround and retinal location (as is evident from Fig. 4b). Post-hoc testing using the `lsmeans`-package (version 2.30.0) for R revealed that center and surround SC are significantly different across all retinal locations:

```
bin_num_str =   one:
  contrast            estimate   SE         df    t.ratio    p.value
  center - surround   -0.660     0.0147     3094   -45.018   <.0001
bin_num_str =   two:
  contrast            estimate   SE          df   t.ratio    p.value
  center - surround   -0.830     0.0343     3073   -24.225   <.0001
bin_num_str =   three:
  contrast            estimate   SE         df    t.ratio    p.value
  center - surround   -0.657     0.0288     3154  -22.801    <.0001
bin_num_str =   five:
  contrast            estimate   SE         df    t.ratio    p.value
  center - surround   -0.542     0.0280     3248  -19.367    <.0001
bin_num_str =   six:
  contrast            estimate   SE         df    t.ratio    p.value
  center - surround   0.231      0.0211     3221  10.912     <.0001
bin_num_str =   seven:
  contrast            estimate   SE         df    t.ratio    p.value
  center - surround   0.347      0.0294     3048  11.788     <.0001
bin_num_str =   eight:
  contrast            estimate   SE         df    t.ratio    p.value
  center - surround   0.258      0.0228     3026  11.293     <.0001
```

For GCL ROIs, the resulting model was fit using *n*=10,804 observations, with *n*=5,788 observations in group cell ID. Running an ANOVA on the model yielded the following results:

Type III Analysis of Variance Table with Satterthwaite's method

	Sum Sq	Mean Sq	NumDF	DenDF	F value	Pr(>F)
cent_surr	441.82	441.82	1	10800	1375.51	<2e-16
bin_number	33.58	33.58	1	10800	104.53	<2e-16
cent_surr:bin	77.65	77.65	1	10800	241.73	<2e-16

The model results indicate that for GCL ROIs there is a significant interaction between center and surround and retinal location (as is evident from Fig. 6b). Post-hoc testing revealed that center and surround SC are significantly different across all retinal locations:

bin_num_str = one:

contrast	estimate	SE	df	t.ratio	p.value
center - surround	-0.6824	0.0456	5558	-14.969	<.0001

bin_num_str = two:

contrast	estimate	SE	df	t.ratio	p.value
center - surround	-0.6281	0.0218	5525	-28.768	<.0001

bin_num_str = three:

contrast	estimate	SE	df	t.ratio	p.value
center - surround	-0.4851	0.0188	5473	-25.853	<.0001

bin_num_str = four:

contrast	estimate	SE	df	t.ratio	p.value
center - surround	-0.5655	0.0383	5626	-14.762	<.0001

bin_num_str = five:

contrast	estimate	SE	df	t.ratio	p.value
center - surround	0-0.9121	0.0615	5972	-14.827	<.0001

bin_num_str = six:

contrast	estimate	SE	df	t.ratio	p.value
center - surround	-0.2191	0.0291	5903	-7.535	<.0001

bin_num_str = seven:

contrast	estimate	SE	df	t.ratio	p.value
center - surround	-0.0653	0.0332	5631	-1.966	<.0001

Generalized Additive Models

We used Generalized Additive Models (GAMs) to analyze the relationship of difference in center and surround SC (SC_{Diff}) and IPL depth; opponency and IPL depth; center SC (SC_{center}) and IPL depth. GAMs extend the generalized linear model by allowing the linear predictors to depend on arbitrary smooth functions of the underlying variables [13]:

$$g(\mu) = \beta_0 + f_1(x_1) + \ldots + f_n(x_n) \quad (4)$$

Here, x_i are the predictor variables, g is a link function and the f_i are smooth functions of the predictor variables.

In practice, we used the `mgcv`-package for R (version 1.8-24) to implement GAMs and perform statistical testing.

SC_{Diff} vs. IPL depth

To model the dependence of SC_{Diff} as a function of IPL depth for dorsal and ventral IPL ROIs (Fig. 4e), we used a Gaussian GAM with factor retinal position ("dv"; 0: ventral; 1: dorsal) and a smooth term for dorsal and ventral retina as a function of IPL depth.

```
SCDiff ~ s(Depth, by = dv, k = 20) + dv + s(exp_num, bs = "re")
```
(5)

We set the basis dimension k=20 to allow for sufficiently "wiggly" smooth terms. Inspection of the model fit indicated that this was high enough (using gam.check). The resulting model was fit using n=2,955 data points and yielded the following results:

```
Parametric coefficients:
```

	Estimate	Std. Error	t value	Pr(>|t|)
(Intercept)	-0.04636	0.01391	-3.33	0.000871
dvV	0.98749	0.01696	58.214	< 2e-16

```
Approximate significance of smooth terms:
```

	edf	Ref. df	F	p-value
s(Depth):dvD	1.0000	1.00	13.45	0.000249
s(Depth):dvV	11.5912	14.04	23.83	< 2e-16

Thus, the smooth terms for dorsal and ventral ROIs are highly significant, indicating non-random variation of SC_{Diff} with IPL depth.

Overall, the model explained 57.2% of the deviance.

Opponency vs. IPL depth

To model the dependence of full-field opponency to light onset as a function of IPL depth for dorsal and ventral IPL ROIs (Fig. 4f), we used a binomial GAM with factor retinal position ("dv"; 0: ventral; 1: dorsal) and a smooth term for dorsal and ventral retina as a function of IPL depth.

```
Opponency ~ s(Depth, by = dv, k = 20) + dv + s(exp_num, bs = "re")
```
(6)

The resulting model was fit using n=2,140 data points and yielded the following results:

```
Parametric coefficients:
                 Estimate   Std. Error  z value   Pr(>|z|)
(Intercept)      -3.3190    0.2179      -15.23    < 2e-16
dvV               3.2472    0.2299      14.13     < 2e-16
Approximate significance of smooth terms:
                 edf        Ref. df     Chi. sq   p-value
s(Depth):dvD     4.2812     5.389       36.33     1.54e-06
s(Depth):dvV     9.4203     11.622      214.39    < 2e-16
```

The smooth terms for dorsal and ventral ROIs are highly significant, indicating non-random variation of number of onset opponent ROIs with IPL depth.

Overall, the model explained 39.2% of the deviance.

Next, we modeled the dependence of full-field opponency to light offset as a function of IPL depth for dorsal and ventral IPL ROIs (Fig. 4f) as described above.

The resulting model was fit using n=2,135 data points and yielded the following results:

```
Parametric coefficients:
                 Estimate   Std. Error  z value   Pr(>|z|)
(Intercept)      -2.5493    0.1533      -16.63    < 2e-16
dvV               4.4377    0.1697      26.12     < 2e-16
Approximate significance of smooth terms:
                 edf        Ref. df     Chi. sq   p-value
s(Depth):dvD     7.3727     9.182       67.165    7.82e-11
s(Depth):dvV     9.8553     12.137      70.106    3.59e-10
```

The smooth terms for dorsal and ventral ROIs are highly significant, indicating non-random variation of number of offset opponent ROIs with IPL depth.

Overall, the model explained 52% of the deviance.

SC_{center} vs. IPL depth

To model the dependence of SC_{center} as a function of IPL depth for dorsal and ventral IPL ROIs (Suppl. Fig. S2b), we used a Gaussian GAM with factor retinal position ("dv"; 0: ventral; 1: dorsal) and a smooth term for dorsal and ventral retina as a function of IPL depth.

SC_{center} ~ s(Depth, by = dv, k = 20) + dv + s(exp_num, bs = "re") (7)

The resulting model was fit using n=3,188 data points and yielded the following results:

```
Parametric coefficients:
                 Estimate   Std. Error  z value   Pr(>|z|)
(Intercept)      0.1837     0.0046      39.68     < 2e-16
dvV              -0.6771    0.0057      -118.22   < 2e-16
```

```
Approximate significance of smooth terms:
                  edf        Ref. df     Chi. sq   p-value
s(Depth):dvD      9.1231     11.23       4.078     4.85e-06
s(Depth):dvV      11.1562    13.57       11.092    < 2e-16
```

The smooth terms for dorsal and ventral ROIs are highly significant, indicating non-random variation of SC_{center} with IPL depth.

Overall, the model explained 82% of the deviance.

SUPPLEMENTARY FIGURES

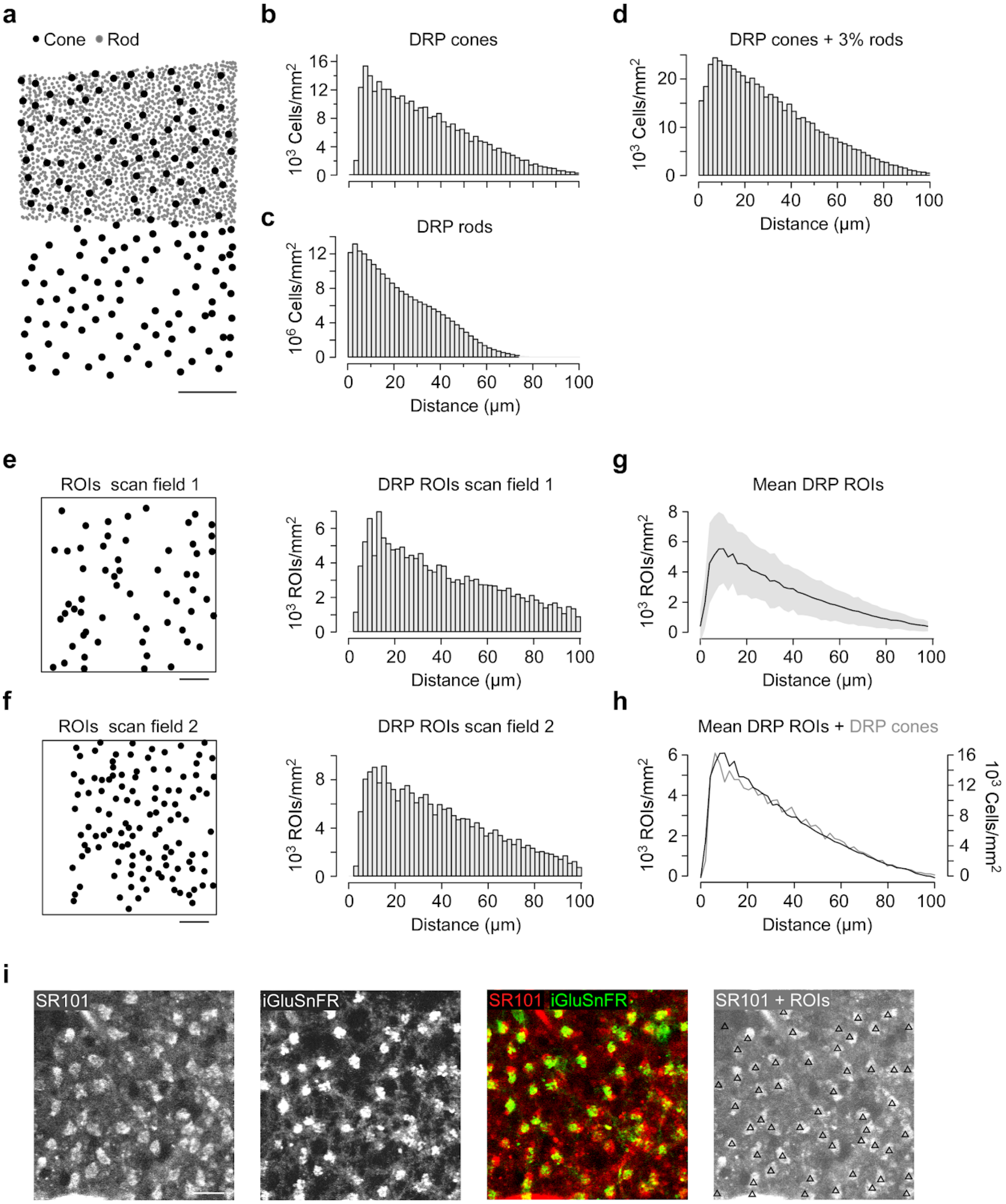

Supplementary Figure 1 | Contribution of rod photoreceptors to glutamate signals in the outer plexiform layer. a, Center-of-mass of cone (black) and rod (grey) axon terminals from electron-microscopy reconstruction [1]. Note that rod axon terminals were only reconstructed for the upper half of the retinal patch. Scale bar: 20 µm. **b, c,** Density recovery profile (DRP; [14]) for cones (b) and rods (c) shown in (a), with 2 µm bins. Note the low number of cells for distances <5 µm for the cone DRP, indicating a regular mosaic array. **d,** DRP of cones from (a) with 3% of rods randomly distributed across the retinal patch. Note that including a low percentage of rods reduces the regularity of the mosaic, indicated by neighboring cells with distances <5 µm. **e,** Exemplary scan field with ROI positions indicated in black and corresponding DRP (right). Scale bar: 20 µm. **f,** Like (e) for a different scan field. **g,** Mean DRP of all OPL scan fields (*n*=52 scan fields, *n*=9 mice) with s.d. shading in grey. **h,** DRP from

anatomical cone data from (b; grey) and mean DRP from (g; black) are not significantly different. $p>0.05$; Chi-squared test. **i,** Sulforhodamine 101 (SR101) labeling of cone axon terminals (left; [15]), iGluSnFR labeling (middle left), overlay image (middle right) and ROI position (black triangles) on top of SR101 labeling (right) for an exemplary scan field. The right image illustrates that functional glutamate release units (ROIs) match anatomically identified cone axon terminals (SR101). Scan field shown corresponds to a representative example. In total, we recorded n=52 scan fields in n=9 mice. Scale bar: 10 µm.

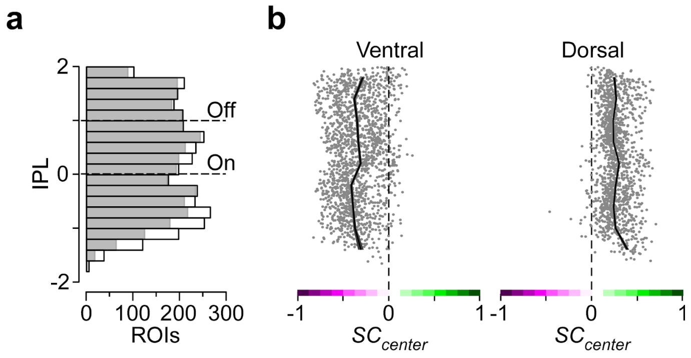

Supplementary Figure 2 | Chromatic glutamate responses across the inner plexiform layer. a, Distribution of all recorded ROIs across the IPL (black), with ROIs that passed our quality criterion indicated in grey (for details, see Methods) and On and Off ChAT bands indicated by dashed line. Bin size: 0.4. **b,** Receptive field (RF) center spectral contrast (SC_{center}) values across the IPL for ROIs located in the ventral (left) and dorsal (right) retina, respectively. For statistics, see Suppl. Information.

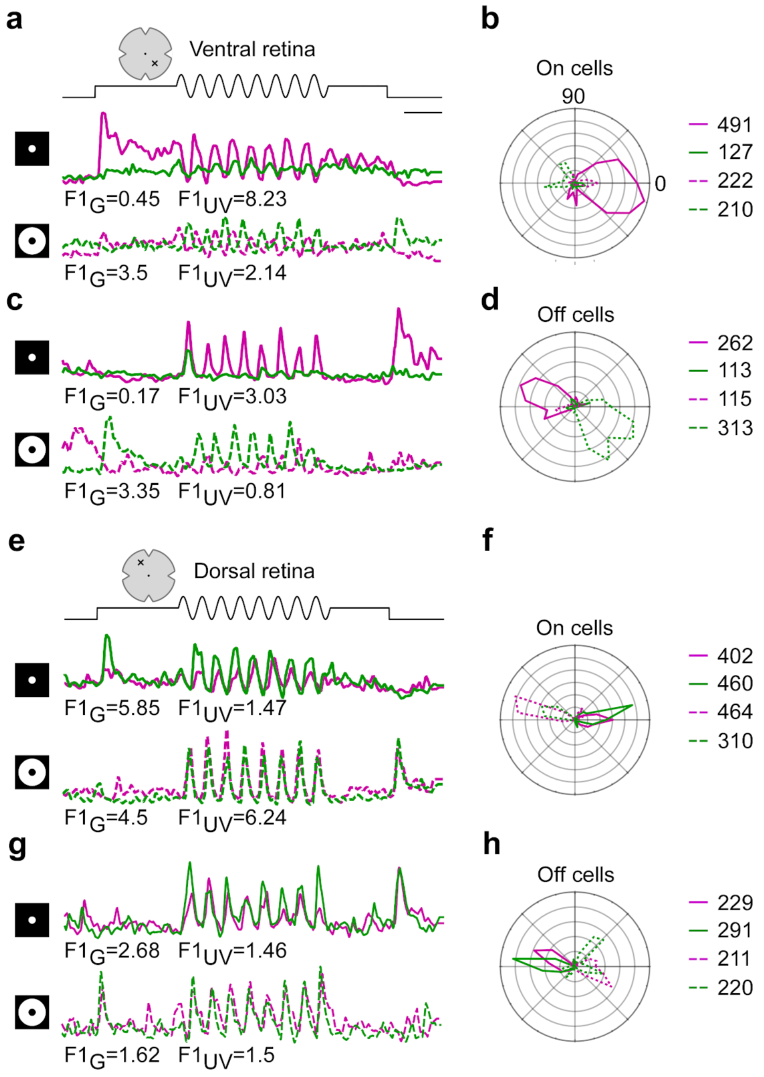

Supplementary Figure 3 | Bipolar cell responses to sinusoidal modulation of green and UV LED. a, Mean glutamate trace (*n*=3 trials) of an exemplary ROI located in the On layer of the IPL in the ventral retina in response to 2 Hz center (top) and surround (bottom, dashed lines) modulation of green and UV LED. Scale bar: 1 s. The fundamental response component (F1) is indicated below the traces. **b,** Polar plot showing distribution of response phases (in degrees) for On cells located in the ventral retina to stimulus shown in (a). Each histogram is normalized according to mean F1 (for details, see Methods). Numbers on the right indicate ROIs used for analysis of each condition. **c,d,** Like (a,b), but for Off cells located in the ventral retina. **e,f,** Like (a,b), but for On cells located in the dorsal retina. **g,h,** Like (a,b), but for Off cells located in the dorsal retina.

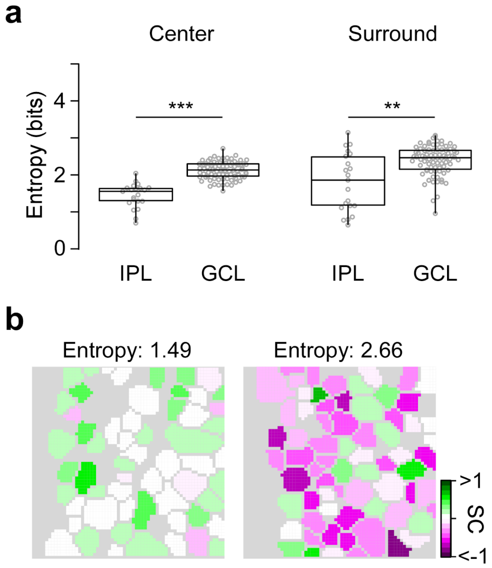

Supplementary Figure 4 | Diversity of chromatic preferences within IPL and GCL scan fields. a, Distribution of field entropy of IPL (*n*=21) and GCL (*n*=82) scan fields using center (left) and surround (right) spectral contrast. High field-entropy indicates high chromatic tuning heterogeneity within single scan fields. Quartile method: Turkey; whisker method: s.d.. **: *p*<0.01; ***: *p*<0.001; Wilcoxon test for unpaired data. **b,** Two exemplary GCL scan fields with low (left) and high (right) entropy.

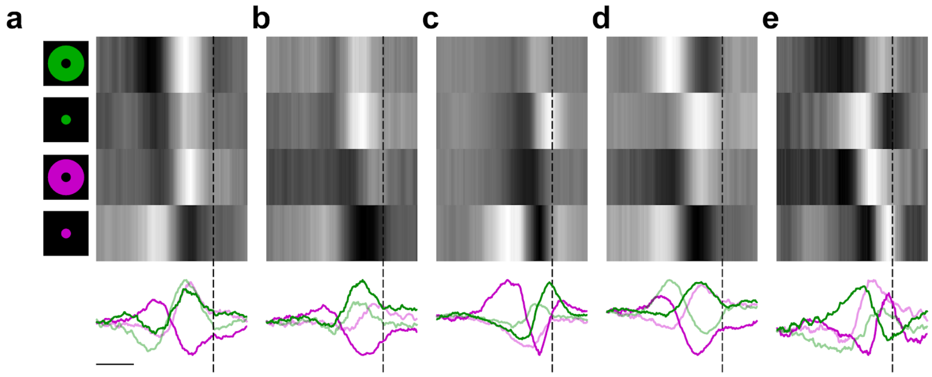

Supplementary Figure 5 | Center-opponent cells in the ganglion cell layer of the mouse. a, Response-triggered stimulus kernels for UV and green center and surround stimulation (top) of exemplary center-opponent GCL cells, with color-coded center (bright) and surround (dim) kernels overlain (bottom, scale bar: 400 ms). Dashed line corresponds to time point of response. **b-e,** Like (a), but for other center-opponent GCL cells. Note the differences in response polarity and kinetics, suggesting that center-opponent GCL cells do not comprise a single functional type.

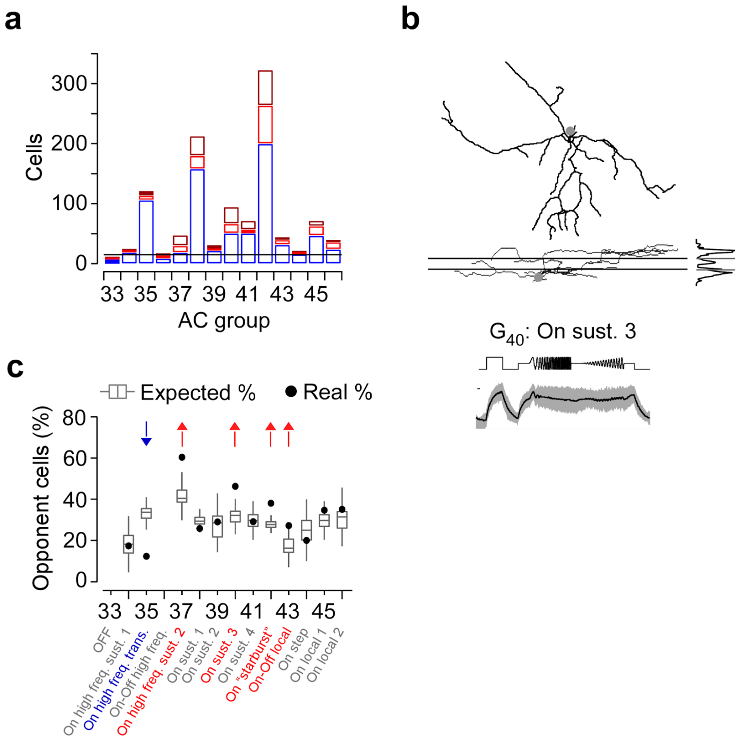

Supplementary Figure 6 | Center-opponent displaced amacrine cells (dAC). a, Distribution of color-opponent (red) and non-opponent (blue) dACs located in the ventral retina. For further analysis, only groups with *n*>10 cells were used. **b,** Dendritic morphologies with stratification profiles of exemplary color-opponent cell assigned to dAC group G_{40}, dye-filled and reconstructed subsequent to imaging experiments. Lines for side-view of morphology and stratification profile indicate On and Off ChAT bands. Mean full-field chirp responses (black, s.d. shading in grey) of the same groups indicated below. **c,** Box plots (quartile method: Turkey; whisker method: s.d.) show distribution of expected percentages of color-opponent cells given center SC and SC_{Diff} values in each group (for details, see Methods). Black circles indicate true percentage of color-opponent cells. Arrows pointing down- and up indicate groups with significantly more and less color-opponent cells than expected, respectively (for cell numbers per group see (a)). G_{33}:-, G_{34}:0.39, G_{35}:0, G_{36}:0.42, G_{37}:0.001, G_{38}:0.089, G_{39}:0.33, G_{40}:0.0006, G_{41}:0.4, G_{42}:0, G_{43}:0.043, G_{44}:0.45, G_{45}:0.1, G_{46}:0.22.

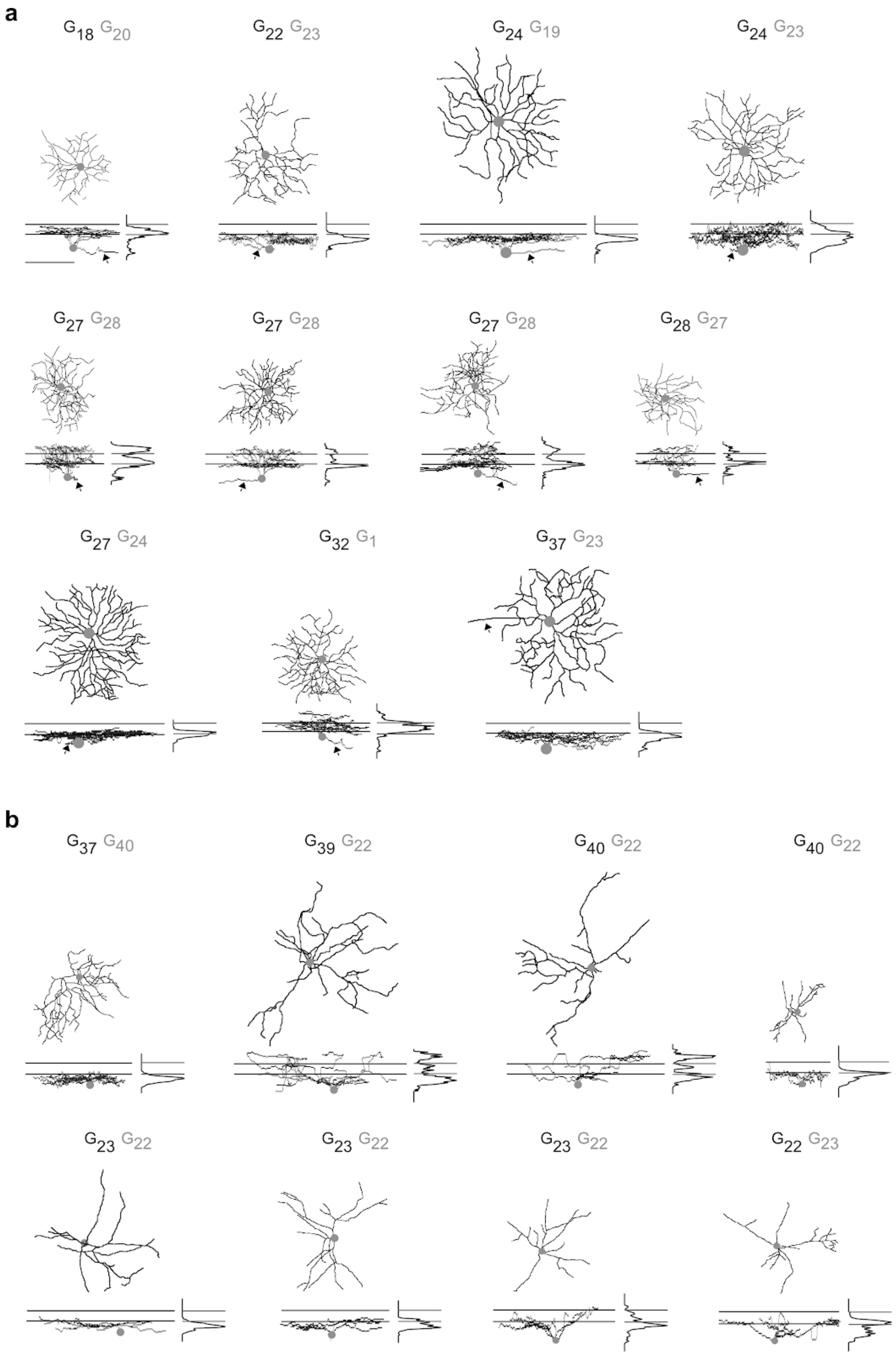

Supplementary Figure 7 | Morphologies of color-opponent GCL cells. **a,** Top-view and side-view of dye-injected and reconstructed color-opponent RGCs identified by the presence of an axon (arrow), with most

(black) and second-most (grey) likely group assignment indicated above morphologies. Scale bar: 100 µm. **b,** Same as (a), but for dACs.

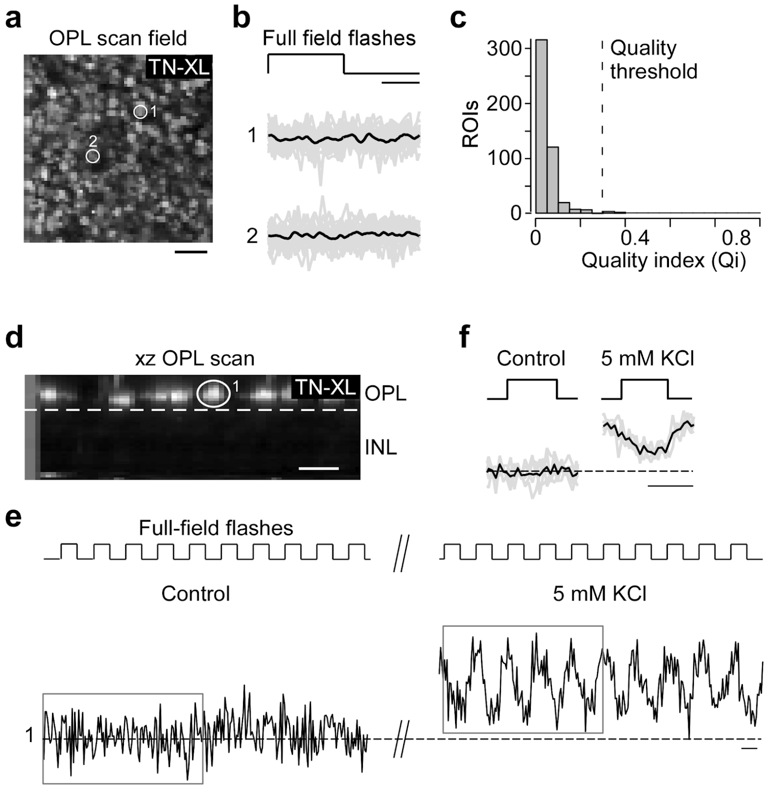

Supplementary Figure 8 | Calcium imaging in the OPL using the biosensor TN-XL. a, Example scan field of cone axon terminals expressing the calcium indicator TN-XL in the outer plexiform layer (OPL) of a whole-mounted HR2.1:TN-XL mouse retina [16], with two axon terminals indicated (circles). Scale bar: 20 μm. **b,** Mean calcium responses (black) with individual trials (grey) to full-field flashes (700 μm in diameter, scale bar: 0.5 s) of the cones indicated in (a). **c,** Histogram of quality indices (Qi) of full-field flash responses (*n*=370 ROIs, *n*=4 scan fields, *n*=4 mice). In contrast to recordings in retinal slices [11,15,17], we did not detect any light-evoked calcium changes in cone axon terminals. Dashed line indicates Qi threshold used for analysis of iGluSnFR data (cf. Fig. 2). **d,** Scan field of a vertical optical slice recording using an electrically tunable lens (ETL), with one axon terminal indicated. Dotted line indicates OPL border. This experiment was only performed for *n*=1 retina dn *n*=1 mouse. Scale bar: 10 μm. **e,** Raw calcium trace of cone shown in (d) in response to full-field flashes for control condition (left; 2.5 mM KCl) and with increased KCl concentration (right; 5 mM KCl). Increasing the extracellular potassium concentration, which is expected to slightly depolarize the cones, recovered their light responses. This suggests that the lack of robust light responses is likely due to the limited dynamic range of TN-XL (discussed in [16]). The difference to the results in slices is probably due to increased laser-evoked activity in the retinal whole-mount preparation (discussed in [18,19]). Dashed line indicates calcium baseline of control condition. Grey rectangles indicate time used for averages shown in (f). Scale bar: 1 s. **f,** Mean calcium trace (black) with individual trials (grey) for control and 5 mM KCl. Scale bar: 0.5 s.

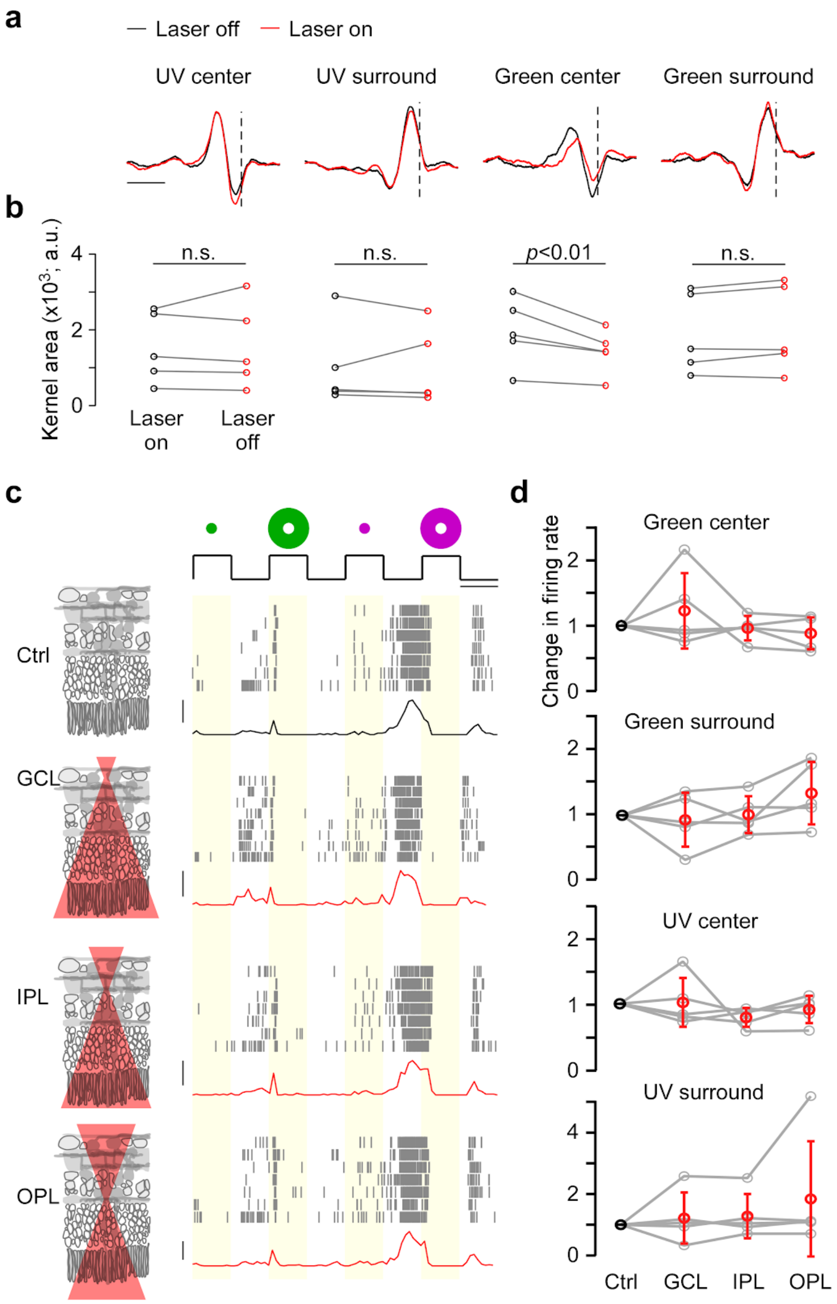

Supplementary Figure 9 | Effect of the two-photon laser on chromatic RGC responses. a, Spike-triggered response kernels for UV and green center and surround stimulation of an exemplary RGC electrically recorded without (black) and with (red) simultaneously scanning the tissue with the two-photon laser. Scale bar: 250 ms. For detailed discussion of laser-induced activity in the retina, see [18,19]. **b,** Areas of UV and green center and surround kernels for *n*=12 electrically recorded RGCs. The two-photon laser consistently reduced the green center component of RGC RFs by ~25%, while it had no detectable effect on UV center or UV and green surround kernels. These results indicate that green-sensitive visual pigments are more strongly activated by the laser than UV-sensitive ones. The effect is restricted to the RF center probably because of the relatively small size of the recording fields. This effect results in an underestimation of the green center component of RFs in our dataset but it does not change the conclusions of this study. Wlcoxon signed-rank test for paired data **c,** Schematic illustrating the position of the laser focus across the retinal layers (left) and spiking activity and spike rate (in Hz, scale bar: 40 Hz) of an exemplary RGC (right) in response to UV and green center and surround flashes (scale bar: 1 s.). Ctrl: without laser. **b, c,** Like (a), but for two additional cells. **d,** Quantification of change in firing rate (*n*=5 cells, *n*=3 animals) for the conditions shown in (a). Note that other than for the flicker stimuli in (a,b), there is no systematic effect of the two-photon laser on RGC flash responses, independent on the focal plane of the laser. Error bars show ± s.d.

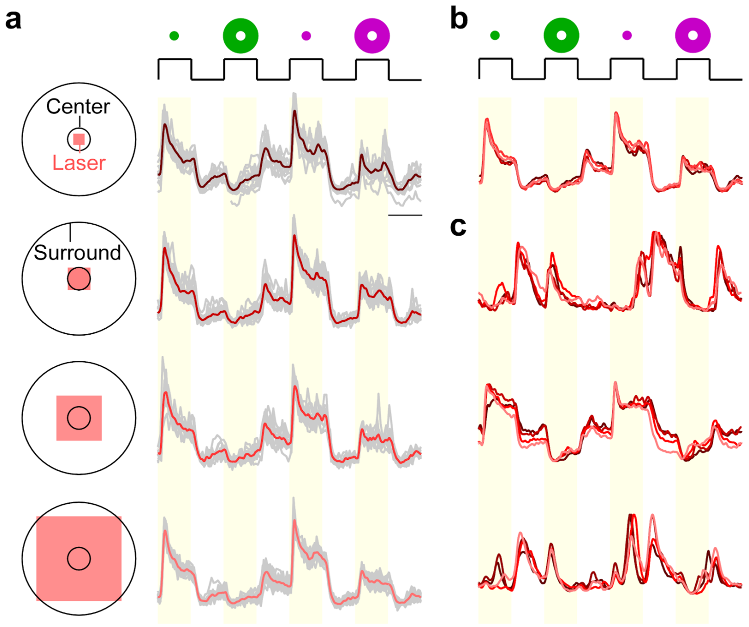

Supplementary Figure 10 | Effect of the two-photon laser on chromatic BC responses. a, Schematic illustrating the size of the 2-photon laser-illuminated area (red) relative to center and surround stimuli and mean glutamate traces (*n*=10 trials in grey) of a small area (30x30 µm) within the On sublamina of the inner plexiform layer (IPL) in response to center and surround UV and green flashes (scale bar: 1 s). **b,** Mean glutamate traces from (a) superimposed. **c,** Like (b), but for three additional scan fields. Increasing the laser-illuminated area had little effect on BC activity, including green surround responses likely mediated by rods. Together with the results shown in Suppl. Fig. S9, this suggests that for our experimental conditions adaptation of rods and cones due to the two-photon laser contributed little to chromatic processing of retinal neurons.

SUPPLEMENTARY REFERENCES

1. Behrens, C., Schubert, T., Haverkamp, S., Euler, T. & Berens, P. Connectivity map of bipolar cells and photoreceptors in the mouse retina. *Elife* **5**, e29941 (2016).
2. Rabl, K., Cadetti, L. & Thoreson, W. B. Kinetics of exocytosis is faster in cones than in rods. *J. Neurosci.* **25**, 4633–4640 (2005).
3. Carter-Dawson, L. D. & LaVail, M. M. Rods and cones in the mouse retina. I. Structural analysis using light and electron microscopy. *J. Comp. Neurol.* **188**, 245–262 (1979).
4. Haverkamp, S., Grünert, U. & Wässle, H. The cone pedicle, a complex synapse in the retina. *Neuron* **27**, 85–95 (2000).
5. Chun, M.-H., Grünert, U., Martin, P. R. & Wässle, H. The synaptic complex of cones in the fovea and in the periphery of the macaque monkey retina. *Vision Res.* **36**, 3383–3395 (1996).
6. Verweij, J., Kamermans, M. & Spekreijse, H. Horizontal cells feed back to cones by shifting the cone calcium-current activation range. *Vision Res.* **36**, 3943–3953 (1996).
7. Burkhardt, D. A. The influence of center-surround antagonism on light adaptation in cones in the retina of the turtle. *Vis. Neurosci.* **12**, 877–885 (1995).
8. Feigenspan, A. *et al.* Expression of connexin36 in cone pedicles and OFF-cone bipolar cells of the mouse retina. *J. Neurosci.* **24**, 3325–3334 (2004).
9. DeVries, S. H., Qi, X., Smith, R., Makous, W. & Sterling, P. Electrical coupling between mammalian cones. *Curr. Biol.* **12**, 1900–1907 (2002).
10. Hornstein, E. P., Verweij, J. & Schnapf, J. L. Electrical coupling between red and green cones in primate retina. *Nat. Neurosci.* **7**, 745–750 (2004).
11. Baden, T. *et al.* A tale of two retinal domains: near-optimal sampling of achromatic contrasts in natural scenes through asymmetric photoreceptor distribution. *Neuron* **80**, 1206–1217 (2013).
12. Bates, D., Mächler, M., Bolker, B. & Walker, S. Fitting Linear Mixed-Effects Models Using lme4. *Journal of Statistical Software, Articles* **67**, 1–48 (2015).

13. Wood, S. N. *Generalized additive models: an introduction with R*. (Chapman and Hall/CRC, 2006).
14. Rodieck, R. W. The density recovery profile: a method for the analysis of points in the plane applicable to retinal studies. *Vis. Neurosci.* **6**, 95–111 (1991).
15. Chapot, C. A. *et al.* Local Signals in Mouse Horizontal Cell Dendrites. *Curr. Biol.* **27**, 3603–3615.e5 (2017).
16. Wei, T. *et al.* Light-driven calcium signals in mouse cone photoreceptors. *J. Neurosci.* **32**, 6981–6994 (2012).
17. Kemmler, R., Schultz, K., Dedek, K., Euler, T. & Schubert, T. Differential regulation of cone calcium signals by different horizontal cell feedback mechanisms in the mouse retina. *J. Neurosci.* **34**, 11826–11843 (2014).
18. Euler, T. *et al.* Eyecup scope—optical recordings of light stimulus-evoked fluorescence signals in the retina. *Pflügers Archiv - European Journal of Physiology* **457**, 1393–1414 (2009).
19. Euler, T., Franke, K. & Baden, T. Studying a Light Sensor with Light: Multiphoton Imaging in the Retina. in *Multiphoton Microscopy* (ed. Hartveit, E.) 225–250 (Springer New York, 2019).
