# Supplementary material for: Neural circuits in the mouse retina support color vision in the upper visual field
Source: Nat Commun. 2020 Jul 13;11:3481. doi: 10.1038/s41467-020-17113-8 (PMC7359335; doi:10.1038/s41467-020-17113-8)
Supplement: Supplementary file 3 — Reporting Summary [file 41467_2020_17113_MOESM3_ESM.pdf]

nature research

Corresponding author(s): Katrin Franke

Last updated by author(s): Apr 1, 2020

Reporting Summary

Nature Research wishes to improve the reproducibility of the work that we publish. This form provides structure for consistency and transparency in reporting. For further information on Nature Research policies, see Authors & Referees and the Editorial Policy Checklist .

Statistics

For all statistical analyses, confirm that the following items are present in the figure legend, table legend, main text, or Methods section.

n/a	Confirmed	
☐	☒	The exact sample size (*n*) for each experimental group/condition, given as a discrete number and unit of measurement
☐	☒	A statement on whether measurements were taken from distinct samples or whether the same sample was measured repeatedly
☐	☒	The statistical test(s) used AND whether they are one- or two-sided<br>*Only common tests should be described solely by name; describe more complex techniques in the Methods section.*
☐	☒	A description of all covariates tested
☐	☒	A description of any assumptions or corrections, such as tests of normality and adjustment for multiple comparisons
☐	☒	A full description of the statistical parameters including central tendency (e.g. means) or other basic estimates (e.g. regression coefficient) AND variation (e.g. standard deviation) or associated estimates of uncertainty (e.g. confidence intervals)
☐	☒	For null hypothesis testing, the test statistic (e.g. *F*, *t*, *r*) with confidence intervals, effect sizes, degrees of freedom and *P* value noted<br>*Give P values as exact values whenever suitable.*
☒	☐	For Bayesian analysis, information on the choice of priors and Markov chain Monte Carlo settings
☐	☒	For hierarchical and complex designs, identification of the appropriate level for tests and full reporting of outcomes
☐	☒	Estimates of effect sizes (e.g. Cohen's *d*, Pearson's *r*), indicating how they were calculated

Our web collection on statistics for biologists contains articles on many of the points above.

Software and code

Policy information about availability of computer code

Data collection	Image acquisition in two-photon glutamate/calcium experiments was performed using custom-made software (ScanM v2.04 by M. Müller and T.Euler) in IGOR Pro 6.3 for Windows (Wavemetrics).<br>Single-cell electrophysiological recordings were performed using an Axopatch 200B amplifier in combination with a Digidata 1440 digitizer (Molecular Devices) at 20kHz.<br>Single cell injections for morphological reconstructions were performed subsequent to two-photon calcium imaging using the buzz function (100 ms pulse) of the MultiClamp 700B software (Molecular Devices).

Data analysis	Preprocessing of the data was performed in IGOR Pro 6.3/7.08 32-bit/8.0 (Wavemetrics): for GCL recordings regions of interests (ROIs) were defined semi-automatically by custom software (as in Baden et al., 2016), for OPL and IPL recordings ROIs were defined automatically by custom correlation-based algorithms (see Franke et al., 2017). Glutamate/calcium traces for each ROI were extracted using custom analysis code based on the image analysis toolbox SARFIA in IGOR Pro (Dorostkar et al., 2010).<br>Custom scripts in IGOR Pro 6.3/7.08 32-bit/8.0 were used in order to compute: glutamate/calcium event-triggered average ("event-triggered stimulus kernels"); the stimulus-triggered average glutamate/calcium event ("stimulus-triggered event kernels"); response quality indices; spectral contrast for estimating chromatic preference; density recovery profiles of OPL ROI masks; anatomical DRPs from an available EM dataset (cf. Suppl. Fig. 1, Behrens et al., 2016); field entropy; full-field opponency; direction selectivity (as in Baden et al., 2016) and the statistical significance of directional tuning using a permutation test (Ecker et al., 2014); cell responses to sinusoidal modulation; identification of functional RGC cluster (as in Baden et al., 2016).<br>Injected cells for morphological reconstructions were traced semi-automatically with the use of the Simple Neurite Tracer plugin implemented in Fiji (https://imagej.net/Simple_Neurite_Tracer). Additionally, to correct any warping of the IPL, the original image stacks were de-warped using custom-written scripts in IGOR Pro 6.3/7.08 32-bit/8.0 (as in Baden et al., 2016).<br>For statistical analysis the following packages were used: the lme4- package (version 3.0.1) for R to implement Linear Mixed-Effects Model to analyze the difference between center and surround SC for OPL, IPL and GCL recordings and perform statistical testing; and the mgcv- package (version 1.8 -24) for R to implement Generalized Additive Models (GAMs) to analyze the relationship of difference in center and surround SC and IPL depth, opponency and IPL depth, center chromatic preference and IPL depth and perform statistical testing.

For manuscripts utilizing custom algorithms or software that are central to the research but not yet described in published literature, software must be made available to editors/reviewers. We strongly encourage code deposition in a community repository (e.g. GitHub). See the Nature Research guidelines for submitting code & software for further information.

Data

Policy information about availability of data

All manuscripts must include a data availability statement. This statement should provide the following information, where applicable:

- Accession codes, unique identifiers, or web links for publicly available datasets
- A list of figures that have associated raw data
- A description of any restrictions on data availability

The dataset that supports the findings of our study and custom-written scripts are available online (data: https://doi.org/10.5281/zenodo.3742765, code: https://github.com/frankelab/retina_color).

Field-specific reporting

Please select the one below that is the best fit for your research. If you are not sure, read the appropriate sections before making your selection.

☒ Life sciences ☐ Behavioural & social sciences ☐ Ecological, evolutionary & environmental sciences

For a reference copy of the document with all sections, see nature.com/documents/nr-reporting-summary-flat.pdf

Life sciences study design

All studies must disclose on these points even when the disclosure is negative.

Sample size	We did not perform prior sample size calculation. As the experiments are hard to do, we simply used all cells we could record in a reasonable time frame. We made sure each condition (e.g.) was sufficiently covered with >3 independent samples.
Data exclusions	We only used ROIs for analysis that passed the quality thresholds as described in the Methods section. Similar quality thresholds were used in previous studies (Baden at al., Nature 2016; Franke et al., Nature 2017).
Replication	We consider each recorded scan field as an independent sample, and all figures reflect $n \geq 3$ biologically independent replicates. The experimental outcomes were consistent across recordings and animals. Experiments were replicated in 9 (Cx57+/+ mice) and 3 (HR2.1:TN-XL mice) mice for OPL recordings, 5 mice for IPL recordings and 23 mice for GCL/RGC recordings.
Randomization	There are no experimenter defined experimental groups in this study, so no randomization was performed. The recorded cells were classified into functional groups based on previous studies (Baden at al., Nature 2016; Franke et al., Nature 2017).
Blinding	Blinding was not relevant because of the exploratory nature of the study (basic research).

Reporting for specific materials, systems and methods

We require information from authors about some types of materials, experimental systems and methods used in many studies. Here, indicate whether each material, system or method listed is relevant to your study. If you are not sure if a list item applies to your research, read the appropriate section before selecting a response.

Materials & experimental systems

n/a	Involved in the study
x	Antibodies
x	Eukaryotic cell lines
x	Palaeontology
	x Animals and other organisms
x	Human research participants
x	Clinical data

Methods

n/a	Involved in the study
x	ChIP-seq
x	Flow cytometry
x	MRI-based neuroimaging

Animals and other organisms

Policy information about studies involving animals; ARRIVE guidelines recommended for reporting animal research

Laboratory animals	Mice from 5 to 18 weeks old of either sex were used for all experimental conditions. OPL recordings: Cx57+/+ (n=9) and HR2.1:TN-XL (n=3) mice. IPL recordings: ChatCre crossbred with Cre-dependent red fluorescent line Ai9tdTomato (JAX 007905) mice (n=5). GCL/RGC recordings: C57Bl/6 (n=14) mice and PvalbCre crossbred with Cre-dependent red fluorescent line Ai9tdTomato (JAX 007905) mice (n=9).
Wild animals	No wild animals were used in this study.
Field-collected samples	No field-collected samples were used in this study.
Ethics oversight	All animals procedures were approved by the governmental review board (Regierungspräsidium Tübingen, Baden-Württemberg, Konrad-Adenauer-Str. 20, 72072 Tübingen, Germany) and performed according to the laws governing animal experimentation issued by the German Governmet.

Note that full information on the approval of the study protocol must also be provided in the manuscript.
